# Bilateral giant open lip schizencephaly with associated cerebral anomalies: a case report

**DOI:** 10.1186/1757-1626-2-7012

**Published:** 2009-04-27

**Authors:** Serhat Avcu, Ã–zkan Ã–zen, Ã–zkan Ãœnal

**Affiliations:** 1 Department of Radiology, YÃ¼zÃ¼ncÃ¼ Yil University School of Medicine, Kazim Karabekir Cad, 65200, Van, Turkey

## Abstract

A nine-month old boy was brought to our hospital with a complaint of growth retardation. On cerebral magnetic resonance imaging examination, giant clefts resulting in the connection of lateral ventricles with subarachnoidal spaces were detected in both cerebral hemispheres, and interpreted as bilateral giant open-lip schizencephaly. Associated anomalies were noted on cerebral magnetic resonance imaging examination. Bilateral thalami were located inferiorly. The caudate nuclei were observed but their configuration was disrupted. The other parts of the basal ganglia including globus pallidi and putamina were absent. We report a case of bilateral giant open-lip schizencephaly with accompanying basal ganglia anomalies.

## Introduction

In 1946, Yakovlev and Wadsworth first described schizencephaly as hemispheric clefts in the region of the primary fissures, infolding of gray matter along the clefts, and associated cerebral malformations, including ventriculomegaly, polymicrogyria, heterotopias, agenesis of the corpus callosum, and absence of the septum pellucidum [1,2]. Currently schizencephaly is described as two types; closed-lip schizencephaly is characterized by gray matter-lined lips that are in contact with each other (type 1). Open-lip schizencephaly has separated lips and a cleft of cerebrospinal fluid, extending to the underlying ventricle (type 2) [1]. Here we report a case of bilateral giant open-lip schizencephaly with accompanying cerebral anomalies.

## Case presentation

A nine-month old Turkish boy was brought to our hospital with a complaint of retardation in growth and development. His parents gave an anamnesis of vomiting that occurred two times a week for the last 4 months, and difficulty in voiding for the last 3 months. The baby could recognize his mother at the 5^th^ month of development. On physical examination, it was seen that the patient could keep his head upright, which was macrocephalic in appearance. The skin overlying the scalp was thinner than normal. All the laboratory examinations were within normal limits. His family history was not significant.

**Figure 1:**
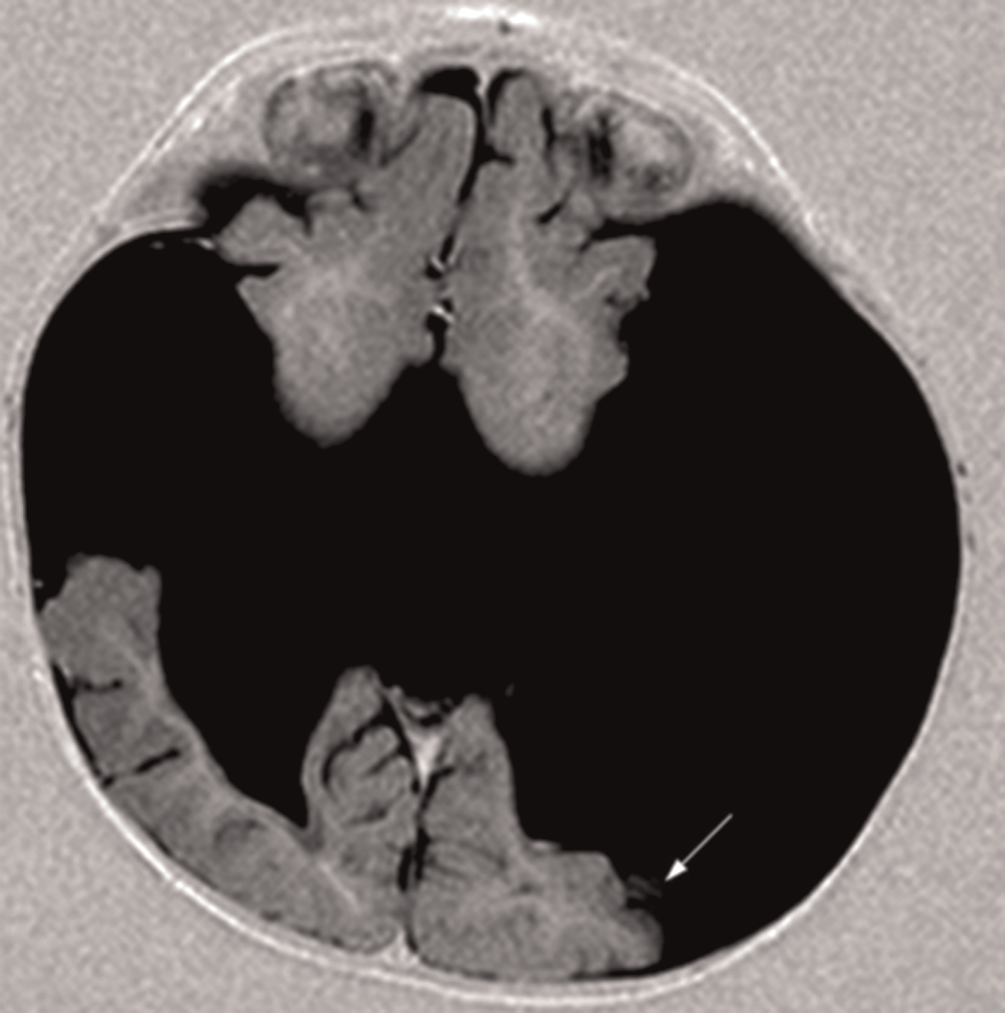
**Polymicrogyric-dysplastic gray-matter surrounding the clefts bilaterally (arrow)**.

**Figure 2:**
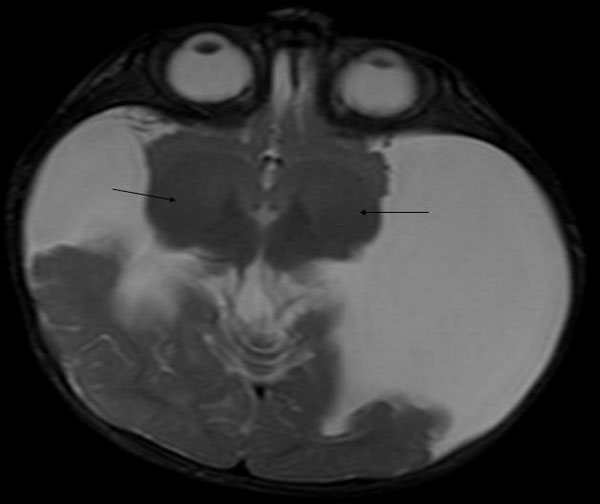
**Axial T2-weighted MRI image showing bilateral inferiorly located thalami (arrows) and giant schizencephaly clefts**.

**Figure 3:**
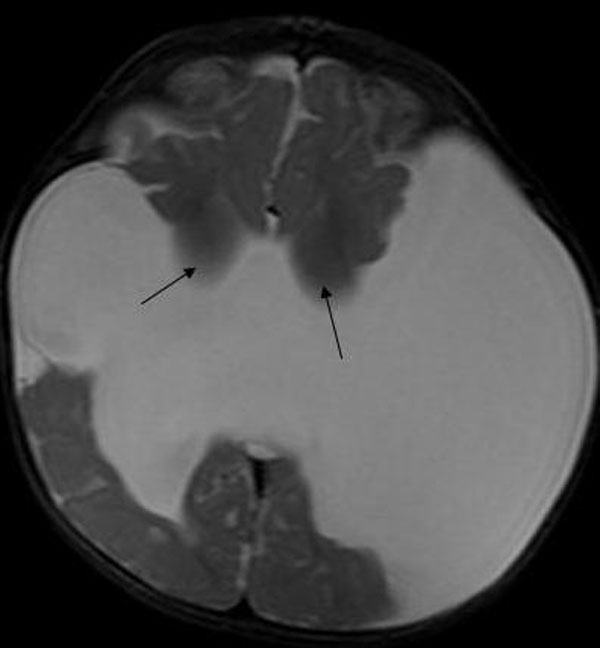
**Structures likely to be the caudate nuclei are observed, but their configuration is disrupted (arrows)**. The other parts of the basal ganglia including globus pallidi and putamina are absent.

On cerebral magnetic resonance imaging (MRI) examination which was performed with 1,5 Tesla Siemens Symphony MRI system, giant clefts resulting in the connection of lateral ventricles with subarachnoidal spaces were detected in both cerebral hemispheres. Polymicrogyric-dysplastic gray-matter was surrounding the clefts bilaterally (Figure 1) which helped us in the diagnosis of schizencephaly. Associated anomalies were noted: bilateral thalami were located inferiorly, and septum pellucidum was absent (Figure 2). Although structures likely to be the caudate nuclei were observed, their configuration was disrupted. The other parts of the basal ganglia including globus pallidi and putamina were absent (Figure 3). The posterior part of the corpus callosum was thinned.

## Discussion

The first description of schizencephaly is as â€œbilateral, almost symmetrical clefts between the pial surface of the cerebral hemisphere and the ependyma of the lateral ventricle that are covered by gray matterâ€�. The schizencephaly clefts are mostly perisylvian or centrally located. Holoprosencephaly, arachnoid cyst, hydranencephaly, and porencephaly are included in the differential diagnosis of schizencephaly. The gray matter covering the clefts, which is mostly polymicrogyric and rarely dysplastic, helps in the differential diagnosis.

The stages of neuronal migration have been completely understood in the past century [2]. At the 8th week of gestation, neuronal migration starts to form the cerebral cortex from the germinal matrix that has been developed as a result of the mitotic activity which has been started at the 7^th^ week of gestation at the subependymal layer of the lateral ventricles. The neuronal migration is induced by radial glial cells [3]. It is claimed that the maturation level of the ependyma that covers the germinal matrix may have an important role in the neuronal migration and maturation [4].

Different theories have been described in the etiology of schizencephaly. Barkovich and Norman have hypothesized a vascular etiology. They proposed the abnormality results from an infarction in an area of the germinal matrix during the seventh week of embryogenesis. One hypothesis is based on vascular compromise during early neuroembryogenesis [9]. Pathologies like infection, metabolic disorders, ischemia, or genetic defects that cause errors in any of the stages of stem cell differentiation, neuronal migration, or cortical organization form cortical anomalies such as lissencephaly, pachygyria, schizencephaly, heterotopia, polymicrogyria, and unilateral megalencephaly [5,6].

The etiology is unclear, although a primary malformation secondary to a neuronal migrational anomaly is considered most likely. Familial cases of schizencephaly have been reported, suggesting a possible genetic origin within a group of neuronal migration disorders. Brunelli et al. [7] have reported heterozygous mutations of the EMX2 gene associated with schizencephaly [8,9]. However, early prenatal injury, such as that associated with drug abuse or abdominal trauma, has also been reported to be associated with schizencephaly, possibly from a vascular insult [11]. Iannetti et al reported cases of clefts resulting from cytomegalovirus infection [12]. Therefore, the appearance of schizencephaly is likely secondary to multiple factors, leading to a final common manifestation of abnormal neuronal migration.

In some of the patients with severe schizencephaly, mutations in â€˜Homeobox EMX2â€™ gene of the 10q2.6 chromosome have been reported [7,8]. Optic nerve hypoplasia, septum pellucidum or corpus callosum agenesis, polymicrogyria, or gray matter heterotopia may accompany schizencephaly [10]. In case of schizencephaly, one should look for accompanying basal ganglia anomalies as well as other cerebral anomalies.

## List of abbreviations

MRI Magnetic resonance imaging.

## Consent

Written informed consent was obtained from the patient for publication of this case report and accompanying images. A copy of the written consent is available for review by the Editor-in-Chief of this journal.

## Competing interests

The authors declare that they have no competing interests.

## Authorsâ€™ contributions

SA, Ã–Ã–, and Ã–Ãœ analyzed and interpreted the patient data regarding the clinical and radiological findings of the patient. All authors were a major contributor in writing the manuscript. All authors read and approved the final manuscript.
